# A randomised bite force study assessing two currently marketed denture adhesive products compared with no‐adhesive control

**DOI:** 10.1002/cre2.182

**Published:** 2019-05-14

**Authors:** Roshan Varghese, Gary R. Burnett, Audrey Souverain, Avinash Patil, Ana G. Gossweiler

**Affiliations:** ^1^ GSK Consumer Healthcare Weybridge UK; ^2^ GSK Consumer Healthcare Nyon Switzerland; ^3^ Department of Statistics Syneos Health Pune India; ^4^ Oral Health Research Institute Indiana University School of Dentistry Indianapolis Indiana

**Keywords:** adhesives, bite force, dentures, edentulous, incisor, randomised controlled trial

## Abstract

Unlike other oral care products, there are limited technologies in the denture adhesive category with the majority based on polymethyl vinyl ether/maleic anhydride (PVM/MA) polymer. Carbomer‐based denture adhesives are less well studied, and there are few clinical studies directly comparing performance of denture adhesives based on different technologies. This single‐centre, randomised, three‐treatment, three‐period, examiner‐blind, crossover study compared a carbomer‐based denture adhesive (Test adhesive) with a PVM/MA‐based adhesive (Reference adhesive) and no adhesive using incisal bite force measurements (area over baseline over 12 hr; AOB_0–12_) in participants with a well‐made and at least moderately well‐fitting complete maxillary denture. Eligible participants were randomised to a treatment sequence and bit on a force transducer with increasing force until their maxillary denture dislodged. This procedure was performed prior to treatment application (baseline) and at 0.5, 1, 3, 6, 9, and 12 hr following application. Forty‐four participants were included in the modified intent‐to‐treat population. AOB_0–12_ favoured both Test adhesive to No adhesive (difference: 2.12 lbs; 95% CI [1.25, 3.00]; *p* < 0.0001) and Reference adhesive to No adhesive (difference: 2.76 lbs; 95% CI [1.89, 3.63]; *p* < 0.0001). There was a numerical difference in AOB_0–12_ for Test versus Reference adhesive (−0.63 lbs; [−1.51, 0.25]); however, this was not statistically significant (*p* = 0.1555). Treatments were generally well tolerated. Both PVM/MA and carbomer‐based denture adhesives demonstrated statistically significantly superior denture retention compared with no adhesive over 12 hr, with no statistically significant difference between adhesives.

## 1 INTRODUCTION

Denture retention in edentulous individuals can be compromised by a number of factors including loss of bone along the residual ridge, neuromuscular changes, and age‐ or medication‐related alterations in saliva quality/quantity (Felton et al., 2011). Denture adhesives are considered to be useful adjuncts to improve management of denture wearing. When properly used, denture adhesives can improve the retention and stability of dentures and help prevent accumulation of food beneath them (Felton et al., 2011; Papadiochou, Emmanouil, & Papadiochos, 2015); however, the duration of effectiveness of adhesive retention is variable and often dependent upon the product formulation (Felton et al., 2011).

Denture adhesives are complex formulations, typically including synthetic hydrophilic polymers that swell when exposed to saliva and adhere to glycoproteins in the oral mucosa; they may also contain antimicrobial agents, binding agents, humectants, flavouring agents, and plasticisers (Kumar et al., 2015). One well‐investigated and marketed denture adhesive technology is based on a combination of polymethyl vinyl ether/maleic anhydride (PVM/MA) and carboxymethyl cellulose (CMC). In this adhesive, the hydrophilic CMC is involved in initial adhesive hydration and is believed responsible for initial adhesive strength when fitted to a denture. As this hydration proceeds, the PVM/MA then hydrates, and a stronger hold develops (Han et al., 2014).

An alternative, less studied, denture adhesive polymer system comprises a carbomer (a partially cross‐linked polyacrylic acid) combined with CMC (Davies, Farr, Hadgraft, & Kellaway, 1991). When exposed to water, carbomer molecules swell with a corresponding increase in viscosity (Singla, Chawla, & Singh, 2000) to form a hydrogel. The mechanism of carbomer mucoadhesion is still ambiguous, although it is hypothesised to be due to partial uncoiling of the polymeric chain, which promotes mechanical entanglement and interaction of polymers with the mucus glycoprotein and the formation of hydrogen bonds with mucosal tissue (Chatterjee, Amalina, Sangupta, & Mandal, 2017; Park & Robinson, 1987).

Although denture adhesives provide multiple benefits, such as increased comfort and preventing food ingress under the denture, a key advantage is to increase denture retention while biting. This property of denture adhesives has been investigated using a number of different methodologies (Howell & Manly, 1948; Kapur, 1967; Tarbet, Boone, & Schmidt, 1980). The maximum bite force until denture dislodgement clinical model is perhaps the most widely used methodology and has been employed successfully to demonstrate efficacy of denture adhesives to improve denture hold from as early as 30 min to up to 12 hr after adhesive application and, in a limited number of studies, to compare retention among various adhesive formulations (Chew, Philips, Boone, & Swartz, 1984; Chew, Boone, Swartz, & Phillips, 1985; Grasso, 2004; Munoz et al., 2012).

To our knowledge, there are no published studies that have compared the clinical performance of carbomer + CMC denture adhesives with the more established PVM/MA + CMC denture adhesives. This bite force study was performed to assess clinical efficacy of a carbomer‐based denture adhesive formulation (Test adhesive) when used with well‐made and at least moderately well‐fitting complete maxillary dentures. The carbomer + CMC Test adhesive was chosen as it had showed good adhesive properties *in vitro* (Data on file). Although the primary objective was to compare incisal bite force until dislodgement with the Test adhesive and no adhesive over 12 hr, exploratory objectives included comparison between the Test adhesive and a PVM/MA‐containing adhesive (Reference adhesive) over 0.5, 1, 3, 6, 9, and 12 hr and between the three groups at all (other) time points. Participants were also questioned about product oozing, flavour and texture, as well as denture fit, comfort, and ease of removal with the study adhesives.

## 2 MATERIALS AND METHODS

This was a randomised, three‐treatment, three‐period, single‐blind (to the bite‐force examiner), crossover study carried out at a U.S.A.‐based clinical research facility and registered at ClinicalTrials.gov (NCT03037307). The protocol was approved by the Indiana University Human Subjects Office (Protocol Number: 1611353632), and the study was conducted in accordance with the Declaration of Helsinki, the International Conference on Harmonisation of Technical Requirements for Registration of Pharmaceuticals for Human Use and local laws and regulations. Four sets of minor amendments were made to the protocol following ethics committee submission, none of which affected study flow or results.

### 2.1 Participants

Healthy participants aged between 18 and 85 years were selected from the study centre's volunteer database. They had a completely edentulous maxillary arch restored with a conventional full acrylic‐based upper complete denture. The maxillary denture was required to be assessed as at least moderately well‐fitting or better at the screening visit (Kapur Index, Olshan Modification; Olshan, Ross, Mankodi, & Melita, 1992) retention score ≥2 (fair to excellent), stability score ≥2 (fair to excellent). If a participant had a partial or fully edentulous mandibular arch, this was permitted to have been restored with a stable partial or complete denture or implant‐supported denture. All dentures were required to be well‐made based on design and construction criteria, including being constructed from an acceptable material, with adequate vertical dimension, freeway space, horizontal occlusal relationships, and border extension; having an acceptable contour and finish; and having acceptable porosity, tissue surfaces, polished surfaces, colour, and thickness.

Participants were excluded if they were pregnant or breastfeeding or had any clinically significant/relevant oral abnormality, oral soft tissue (OST) finding, or severe chronic disease requiring hospitalisation or any other condition that could affect study participation; an incisal bite relation that could affect bite force measurements; severe dry mouth; a cardiac pacemaker implant; diabetes mellitus requiring insulin; participated in another clinical study or received an investigational drug within 30 days of screening; taken/were taking a bisphosphonate or were receiving daily doses of a medication that might interfere with the study; a known/suspected allergy/intolerance to study materials or ingredients.

If participants typically used denture adhesives to stabilise their dentures, they were permitted to continue this during the washout periods between study visits but could not change their routine during the study. Participants reported to the clinic on treatment visit days without the presence of denture adhesive in either their maxillary or mandibular denture. Participants were not allowed to consume any food or liquid for an hour before the treatment visit, except for small sips of water to take medications. On test days, standardised meals were provided at the study centre, and liquid intake was restricted. Smoking, including e‐cigarettes, and the use of chewing tobacco or other tobacco products were prohibited for the screening and test visits. Participants were not permitted to chew gum throughout the study period.

### 2.2 Clinical procedures

**Figure 1:**
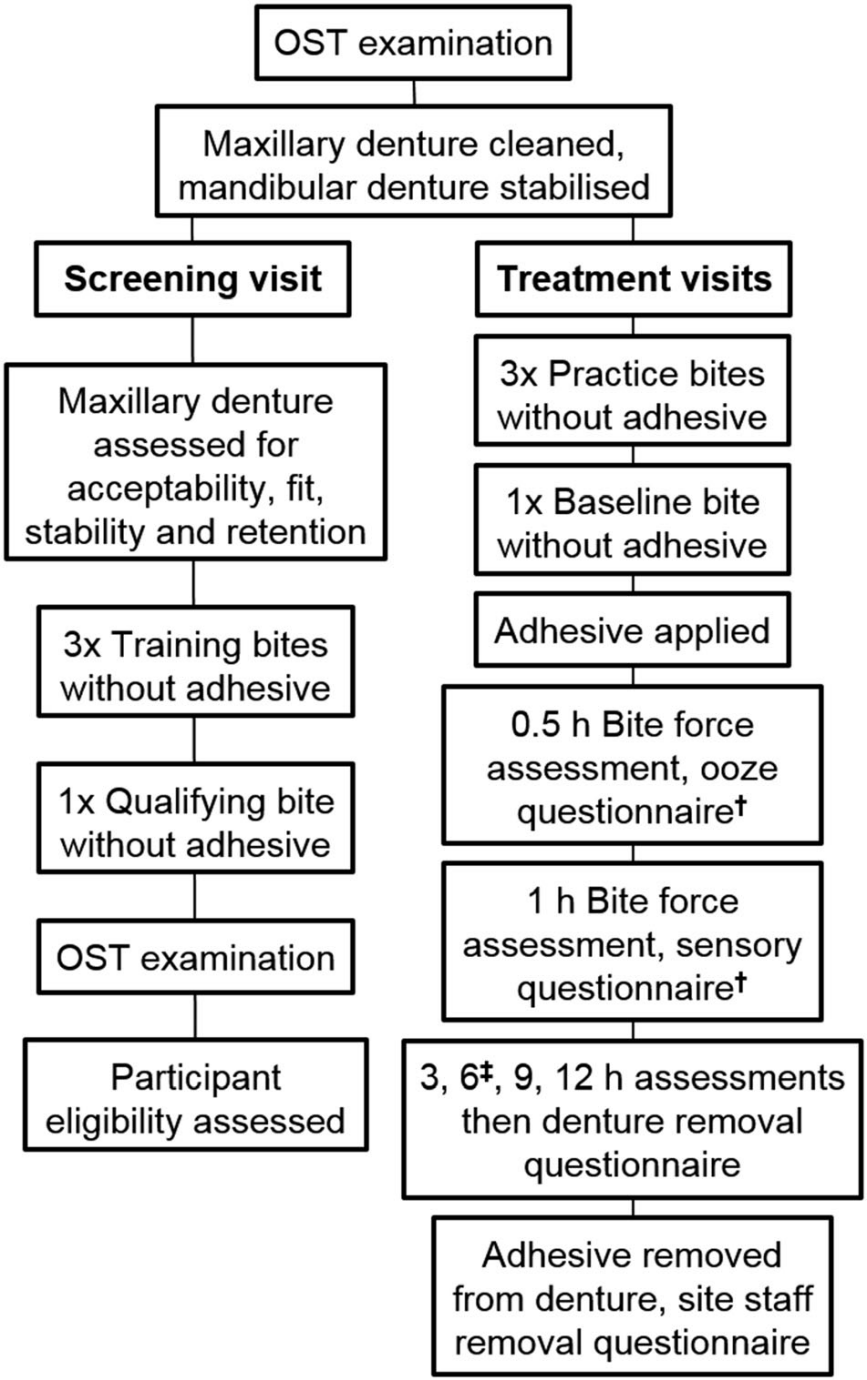
Study procedures **^†^**Ooze questionnaire administered to participants immediately after 0.5‐hr assessment; **^‡^**Standardised lunch given after 6‐hr assessment oral soft tissue, oral soft tissue examination

Participants were required to complete four study visits: screening (Visit 1), then Treatment Visits 2, 3, and 4. A washout period of at least 24 hr was scheduled between each treatment visit to minimise the possibility of carryover effects from study treatments or procedures. Clinical procedures for screening and treatment visits are summarised in Figure 1.

At the screening visit, participants gave written informed consent to participate in the study and were evaluated for eligibility. An OST examination was performed. Dentures were cleaned by site staff using Polident^®^ Dentu‐Crème Denture Cleansing Paste (GSK Consumer Healthcare, Brentford, UK) and Oral B^®^ denture brushes (Procter & Gamble, Cincinnati, OH, USA). If the study participant had a mandibular denture, this was stabilised with denture adhesive (Super Poligrip^®^ Free Adhesive Cream; GSK Consumer Healthcare; USA marketplace) if deemed necessary by the examiner, according to the product label instructions prior to reinsertion. The maxillary denture was inserted without adhesive, then the examiner recorded triplicate bite force until dislodgement measurements (training bites).

The incisal bite force required to dislodge the maxillary denture was assessed using a calibrated bite force transducer system, composed of two plates embedded with a strain gauge that measures displacement of the maxillary denture during biting (Howell & Manly, 1948; Munoz et al., 2012). Here, the examiner inserted the force transducer into the participant's mouth, ensuring correct anterior denture tooth placement on the bite force transducer. The examiner then instructed the participant to bite with increasing force until they felt movement on the maxillary denture, at which time the participant released the bite plate. To minimise interexaminer variability, the same examiner performed all bite force assessments.

Eligible participants were required to have a maxillary incisal bite force measurement (without adhesive) of ≤9 lbs (40.0 N) at the screening visit and prior to treatment application (baseline) at all test visits. At least two of four qualifying bite‐force measurements at screening needed to be reproducible (±2 lbs [8.9 N]).

At Visit 2, dentures were cleaned as described above, and an OST examination performed. The maxillary denture was inserted without adhesive; if present, mandibular dentures were inserted using Super Poligrip Free Adhesive Cream to stabilise. Participants with readings within ±2 lbs for one of the three practice bites and the baseline bite, and with a baseline incisal bite force ≤9 lbs, continued on the study and were randomised to a specific study product order according to a predetermined schedule generated by the Biostatistics Department of the study sponsor using a Williams Square layout.

The three treatment groups were as follows:

- Test adhesive: Protefix^®^ Denture Adhesive, Crème Mint (Queisser Pharma; Flensburg, Germany; Germany marketplace). Ingredients: CMC, carbomer, paraffin, petroleum jelly, silica, wax, flavour, colour, preservative;
- Reference adhesive: Super Poligrip Free Adhesive Cream. Ingredients: PVM/MA (sodium‐calcium mixed partial salt), CMC, petrolatum, mineral oil;
- No adhesive.

Both adhesives were applied in a pattern and manner consistent with the product labels and application instructions. The Test adhesive (1.00 [±0.05] g) was applied by study staff to the clean wet maxillary denture fit surface; the Reference adhesive (1.00 [±0.05] g) was applied to the clean dry maxillary denture fit surface. Dentures were weighed before and after application to ensure the weight of adhesive was correct. The participant positioned the denture in their mouth. For participants in the No adhesive group, the denture was cleaned and dried, then inserted by the participant. Incisal bite force until dislodgement was measured 0.5, 1, 3, 6, 9, and 12 hr after application of the study treatment and/or denture insertion.

Only single applications of the study treatments to the maxillary denture were permitted on each test day. Super Poligrip Free Denture Adhesive Cream was used to stabilise the lower denture to enable the bite force measurements to be performed; this was reapplied to the lower denture up to two times each test day if the investigator deemed it necessary for accurate bite force measurements. Product packaging was overwrapped in white vinyl to mask its identity. To ensure that the examiners remained blind to study allocation, application of denture adhesive was performed in a separate area, and participants were instructed not to disclose whether or not adhesive had been applied.

After the 0.5‐hr measurement, participants allocated to Reference or Test adhesive groups recorded how long they experienced oozing of adhesive around the denture after insertion. After the 1‐hr measurement, participants completed questionnaires relating to the flavour/texture of the adhesive and denture fit/comfort. After the 12‐hr measurement, participants completed questionnaires relating to ease of denture removal, comfort, and coverage of the adhesive and how easy it was to squeeze from the tube (having been provided with a tube to use at this time point). The study staff also completed a questionnaire on how easy it was to remove the adhesive from the denture. Details of the questionnaires are given in Supporting Information (Appendix S1). Posttreatment OST examinations were performed, then dentures were returned to participants. All study site staff who were involved in the collection of bite force and OST examination data were blinded to the distribution and completion of the questionnaires. Visits 3 and 4 proceeded as for Visit 2.

### 2.3 Safety

OST abnormalities and adverse events (AEs) were reported from the end of the OST examination at screening until 5 days after last administration of the study product. Clinical judgement was exercised by the investigator to assess the relationship between the study treatment and the occurrence of each AE, with intensity graded as mild, moderate, or severe.

### 2.4 Statistical analysis

The efficacy analysis was performed on a modified intent‐to‐treat (mITT) population, defined as all participants who were randomised and had at least one postbaseline assessment of efficacy. The per protocol population was a subset of the mITT population where participants with a protocol violation deemed to affect efficacy assessments in all study periods were excluded. The safety population included all participants who were randomised and received the study treatment at least once during the study. Statistical analysis was carried out using SAS v9.2 (SAS Institute Inc., Cary, NC, USA).

Sufficient potential participants were screened so that approximately 45 could be randomised and at least 42 evaluable participants would complete all treatment periods. This sample size was calculated to provide 90% power to demonstrate study success, defined as fulfilment of both the validation objective and the primary objective. The clinically relevant difference detectable was 2.30 lbs for area over baseline between 0 and 12 hr (AOB_0–12_), using two‐sided *t* tests with a 5% significance level, assuming a residual standard deviation (SD; square root of within mean square error) of 2.83 lbs. The estimate of residual SD was obtained as the higher of the observed variability from two previous bite force studies (Jose et al., 2018; Data on file).

The primary efficacy variable was the incisal bite force until denture dislodgement AOB_0–12_. The area under the curve (AUC_0–t_; where t = timepoint) for bite‐force time was calculated using the trapezoidal method. AOB_0–t_ was then calculated as [AUC_0–t_/t] minus baseline bite force value. This transformation returned the measurement to the same scale as the original observations while also looking at the average improvement in bite force AOB by subtracting the baseline value. Linear interpolation was used in the case of missing values. If more than one assessment was missing over the 12 hr assessment period, or if the 12 hr value or baseline value were missing, AOB was set to missing. Higher values of AOB are indicative of a stronger bite force.

An analysis of covariance model was used with AOB values as the response, with fixed effect factors for treatment group and period with participant‐level baseline, and period‐level baseline minus participant‐level baseline as covariates. Participant was included as a random effect. From this model, between‐treatment differences, 95% confidence intervals (CI) and *p* values were provided. All tests were conducted at the two‐sided 5% significance level.

The validity efficacy endpoint was the difference in incisal bite force AOB_0–12_ between the Reference adhesive and No adhesive groups. Demonstrating study validity (*p* < 0.05 for Reference versus No adhesive) was a prerequisite to performing all other treatment comparisons. No further significance testing would be performed if the initial validation step was not achieved. The reason for this validation step was to ensure that the study methodology had been performed as expected, the superior performance of the Reference adhesive over No adhesive having been demonstrated previously (Jose et al., 2018).

The primary efficacy endpoint was the difference in incisal bite force AOB_0–12_ between the Test adhesive and No adhesive groups. Exploratory endpoints included the difference in incisal bite force AOB_0–12_ between the Test adhesive and the Reference adhesive groups. In addition, the incisal bite force AOBs over 0.5, 1, 3, 6, and 9 hr, respectively, for Reference adhesive versus No adhesive, Test adhesive versus No adhesive, and Test adhesive versus Reference adhesive were included as exploratory endpoints, using the AOB calculation (modified according to time interval) and analysis of covariance analysis previously described. The assumptions of normality and homogeneity of variance were investigated for all parametric analyses and considered to be satisfied for validity of primary and exploratory analyses.

Data from the participant‐ and site staff‐completed questionnaires were tabulated and presented using descriptive statistics.

## 3 RESULTS

**Figure 2:**
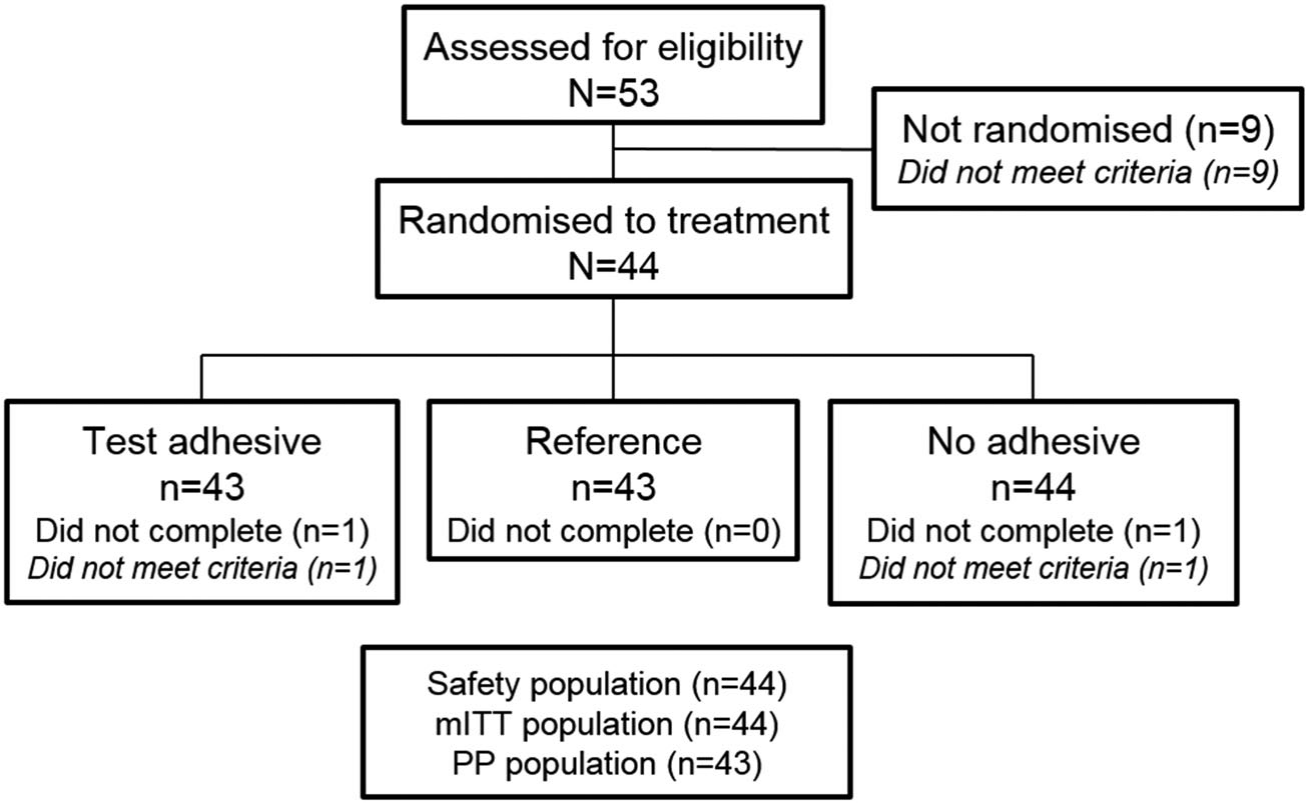
Study flow

The first participant was enrolled in February 2017; the final participant completed the study in May 2017. A total of 53 potential participants were screened; of these, 44 were randomised to treatment and were included in the safety and mITT populations, and 42 completed the study (Figure 2). Of the 44 participants in the safety population, age ranged from 47 to 83 years (mean 67.0 [SD 9.39]) and 59.1% were female. Twenty‐seven participants (64.1%) were black/African American, and 17 participants (38.6%) were white. The weight of adhesive applied for each denture adhesive treatment was within the tolerance specified (1 ± 0.05 g).

### 3.1 Incisal bite force

**Figure 3:**
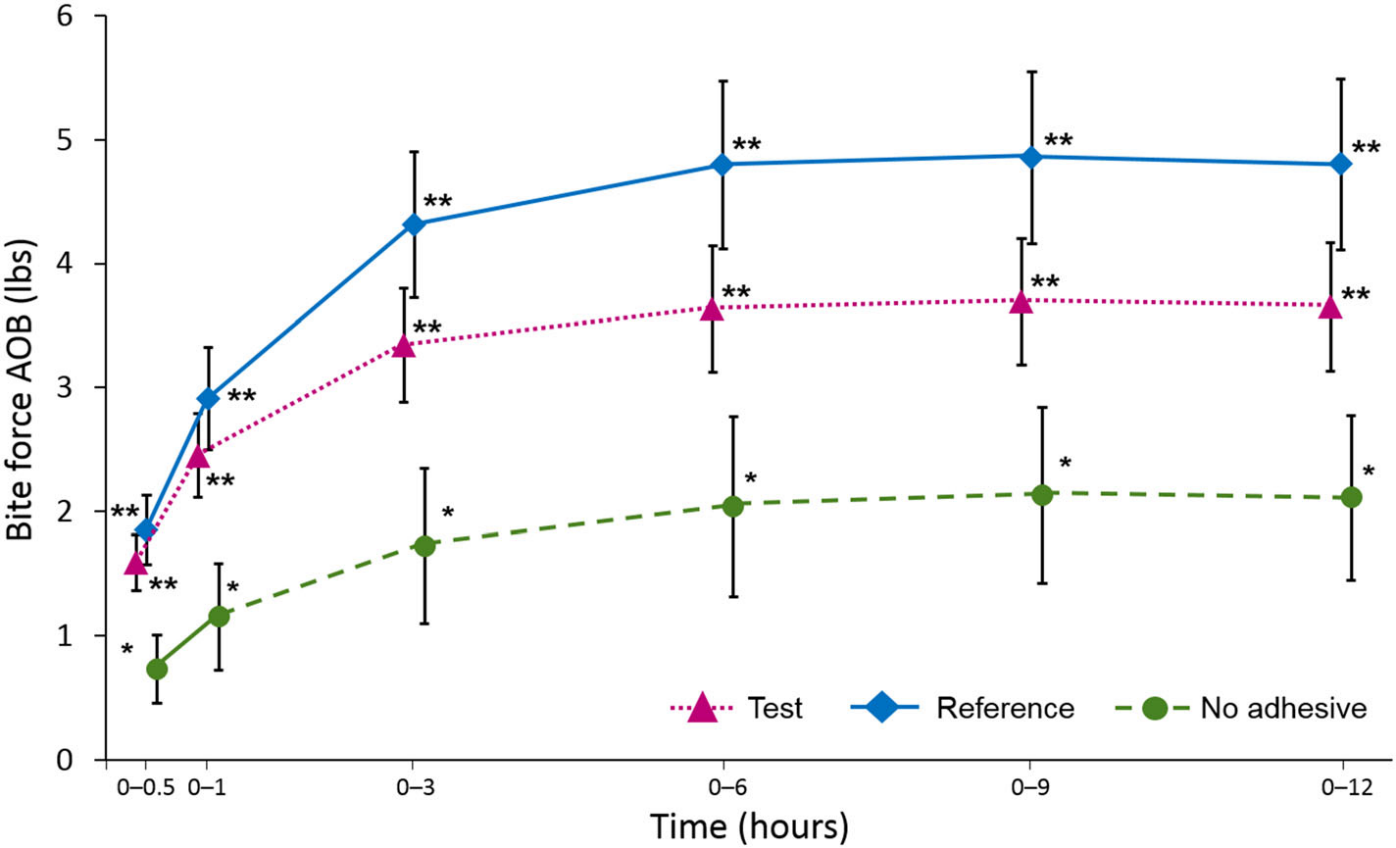
Mean incisal bite force until dislodgement area over baseline (lbs; ±SE) over time (modified Intent‐to‐Treat population) Higher values are more favourable; data points have been offset for clarity; *p < 0.01 versus 0; **p < 0.0001 versus 0

Mean incisal bite force until dislodgement AOB over time for each study group is shown in Figure 3. A statistically significant difference was observed between the Reference adhesive and No adhesive groups in favour of the former at all AOB time points (difference in AOB_0–12_: 2.76 lbs; 95% CI [1.89, 3.63]; *p* < 0.0001; Table 1), demonstrating study validity. Similarly, statistically significant differences were observed between the Test adhesive and No adhesive in incisal bite force until denture dislodgement at all time points in favour of the Test adhesive (difference in AOB_0–12_: 2.12 lbs; 95% CI [1.25, 3.00]; *p* < 0.0001; Table 1). There were numerical differences between the Test and Reference adhesives in favour of the Reference adhesive at all time points, but none of these comparisons were of statistical significance (Table 1).

**Table 1:** Between‐treatment difference in incisal bite force until denture dislodgement area over baseline over different time intervals (modified Intent‐to‐Treat population)

AOB	Treatment difference (lbs)^a^^,^ b (95% confidence intervals) p value		
	Reference adhesive vs. No adhesive	Test adhesive vs. No adhesive	Test adhesive vs. Reference adhesive
AOB_0–12_	2.76 [1.89, 3.63] ***p* < 0.0001**	2.12 [1.25, 3.00] ***p* < 0.0001**	−0.63 [−1.51, 0.25] *p* = 0.1555
AOB_0–9_	2.78 [1.86, 3.70] ***p* < 0.0001**	2.15 [1.23, 3.08] ***p* < 0.0001**	−0.62 [−1.55, 0.30] *p* = 0.1846
AOB_0–6_	2.81 [1.80, 3.81] ***p* < 0.0001**	2.13 [1.12, 3.15] ***p* < 0.0001**	−0.67 [−1.69, 0.34] *p* = 0.1902
AOB_0–3_	2.63 [1.57, 3.69] ***p* < 0.0001**	1.97 [0.90, 3.04] ***p* = 0.0004**	−0.66 [−1.73, 0.42] *p* = 0.2263
AOB_0–1_	1.79 [1.00, 2.59] ***p* < 0.0001**	1.49 [0.68, 2.29] ***p* = 0.0004**	−0.31 [−1.11, 0.50] *p* = 0.4526
AOB_0–0.5_	1.14 [0.63, 1.65] ***p* < 0.0001**	0.99 [0.47, 1.50] ***p* = 0.0003**	−0.15 [−0.66, 0.37] *p* = 0.5717

*Note*. AOB: area over baseline. *p* values in bold indicate statistical significance (*p* < 0.05).

a From ANCOVA with period and treatment group as fixed effects, and participant‐level and period‐level pre‐treatment baseline bite force (parameterized as period‐level minus participant‐level) as covariates. Participant was included as random effect.

b Difference is first‐named treatment minus second‐named treatment; a positive difference favours the first named treatment.

### 3.2 Participant questionnaires

**Figure 4:**
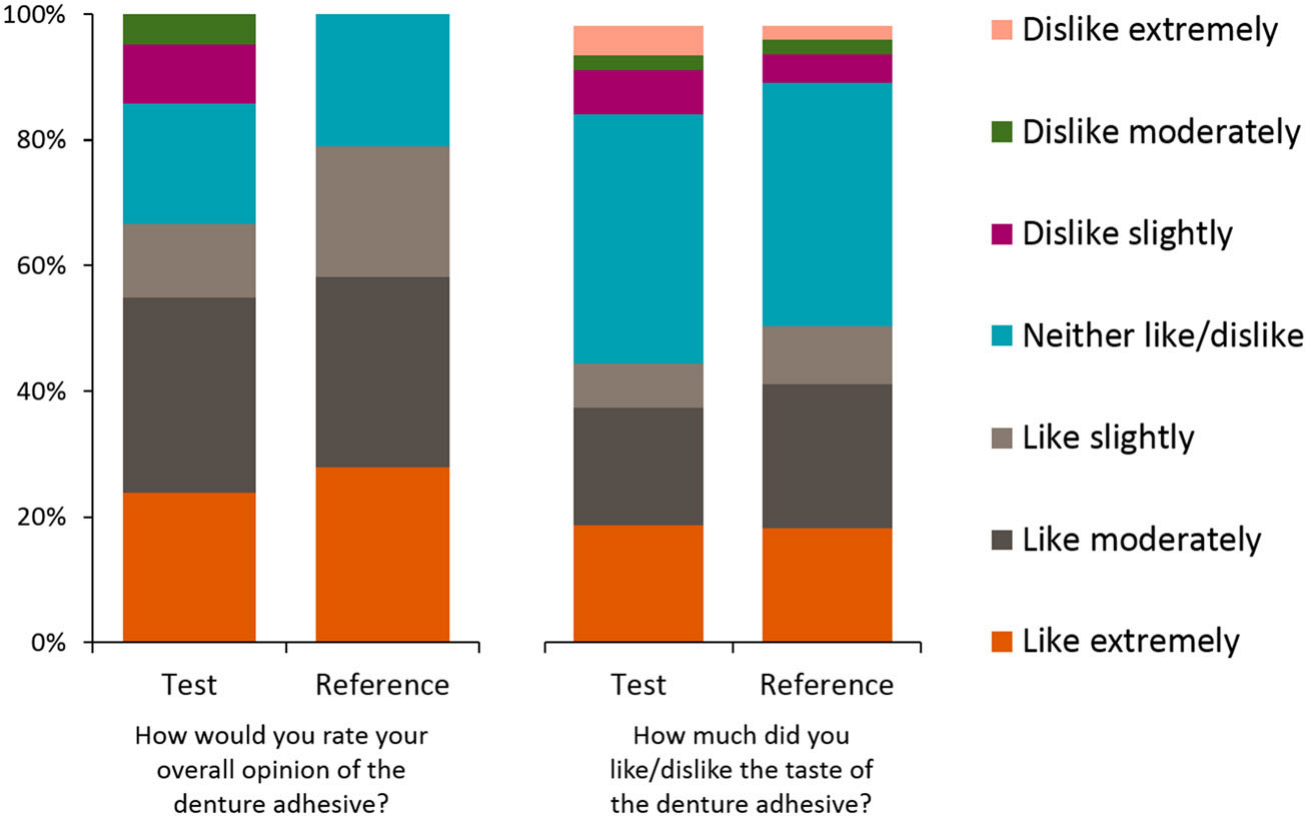
Responses to questionnaires regarding overall opinion and taste of denture adhesive (modified Intent‐to‐Treat population)

**Figure 5:**
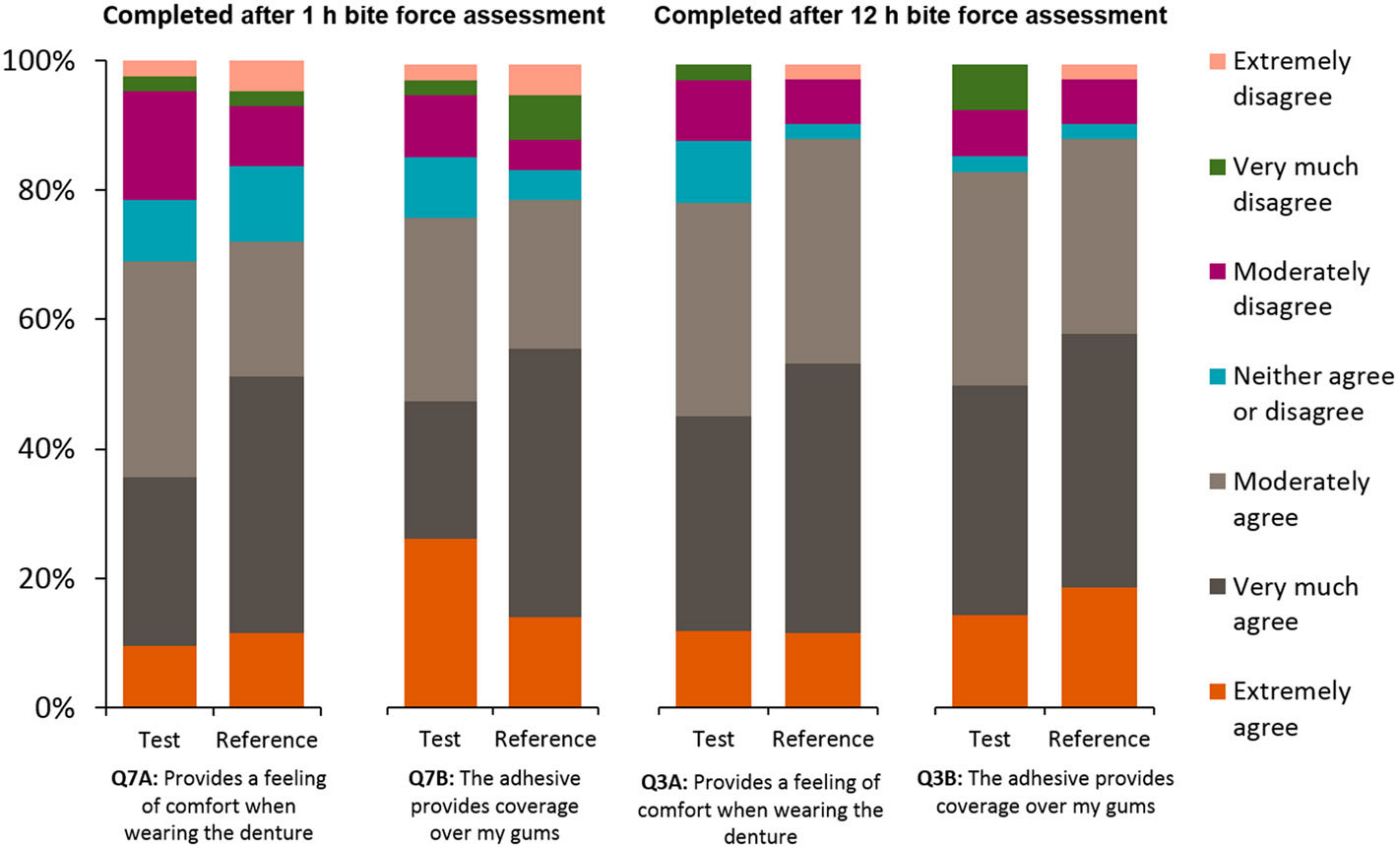
Responses to questionnaires regarding denture adhesive comfort and coverage (modified Intent‐to‐Treat population)

The majority of participants reported they did not experience excessive denture adhesive ooze from underneath the denture with either denture adhesive (Table S1). Questionnaire responses demonstrated no clear notable differences between the Reference and Test adhesives in terms of flavour/texture of the adhesive (Figures 4 and S1) or denture fit/comfort (Figures 5 and S1), although the Reference adhesive did rank slightly higher in most categories. Overall, it was rated easier to remove the dentures following use of the Test adhesive than the Reference adhesive (7.1% of those using the Test adhesive said that it was “Not at all easy” to remove the denture compared with 20.9% of those using the Reference adhesive). The Test adhesive was ranked as “slightly easier” to squeeze from the tube and was “slightly easier” to remove from the gums (Figure S2). The study staff questionnaire response showed that residual Test adhesive was “slightly easier” to remove from the denture than the Reference adhesive (Figure S2).

### 3.3 Safety

Five TEAEs were reported in four participants (9.1%) over the study period. Three TEAEs were recorded as oral events in the Reference adhesive group (medical device pain, gingival erythema, and oral mucosal erythema; all considered treatment related), as was one in the Test adhesive group (mouth injury; not considered treatment related). One non‐oral TEAE occurred in the Test adhesive group (nasopharyngitis; not considered treatment related). All events were mild in intensity, except for the mouth injury event, which was moderate in intensity. All events resolved by study completion, and none resulted in a participant being withdrawn from the study. No serious AEs or incidents were reported.

## 4 DISCUSSION

When properly used, denture adhesives can improve the retention and stability of dentures, help seal out food particles from accumulating beneath the dentures, even in well‐fitting dentures (Felton et al., 2011), and act as a cushion under a complete denture, reducing transmission of pressure and friction to underlying mucosa (Abdelmalek & Michael, 1978). In a pilot study, use of a denture adhesive increased study participants' ability to manage removable full dentures and also enhanced oral health‐related quality of life (Nicolas, Veyrune, & Lassauzay, 2010). Despite this, use of denture adhesives is relatively low (Papadiochou et al., 2015).

This current study investigated denture retention over a 12‐hr period following use of marketed denture adhesives with formulations based on different polymers, as compared with No adhesive. This is one of the first studies to directly compare such adhesives. A “No adhesive” group was included as the majority of denture users do not usually use a denture adhesive (Papadiochou et al., 2015). Study validity was demonstrated by the statistically significant superiority in denture retention as measured by bite force AOB_0–12_ with the Reference adhesive compared with No adhesive, indicating that the clinical model worked as expected. The efficacy criterion for success was also met in that denture retention as measured by bite force AOB_0–12_ for the Test adhesive was statistically significantly superior to No adhesive. Furthermore, both the Test and Reference adhesives demonstrated statistically significantly greater denture retention at all other time intervals (AOB_0–0.5 to 9_) compared with No adhesive. There were no statistically significant differences between the Test and Reference adhesives at any time interval although there was a clear trend with the Reference adhesive having a numerically higher AOB at all time intervals.

This study successfully demonstrated that an adhesive based on carbomer instead of PVM/MA can also deliver improved denture retention up to 12 hr relative to No adhesive. This shows that alternative polymer combinations to the well‐tested PVM/MA plus CMC combination can be useful in promoting denture retention and therefore warrant further investigation and development. The carbomer may also offer differentiated benefits based on its known ability to form hydrogels, which can help form a layer between the denture and the gum surface and thereby potentially provide cushioning from the stresses that the denture can exert on the underlying tissue surfaces.

Other important aspects of a denture adhesive are its ease of use and its “mouth feel” or how an individual experiences the taste and texture of the product. In this study, there were no clear differences in the ooze of the adhesive or sensory preferences expressed by participants in favour of either adhesive. The Test adhesive was slightly easier to squeeze from the tube and remove from the gums and the denture than the Reference adhesive; however, no statistical inference was performed on these comparisons, so definitive conclusions cannot be drawn. Further studies are required to qualify other benefits that carbomer technology may offer compared with PVM/MA‐based options in terms of denture wearer usage experience.

In conclusion, the denture adhesive based on a carbomer formulation and the denture adhesive based on PVM/MA both demonstrated statistically significantly superior denture retention than No adhesive over 12 hr, with no statistically significant difference between adhesives. The differences in the products concerning participant perception of ease‐of‐use and organoleptic preferences may allow greater consumer choice of denture adhesive, potentially increasing adoption in individuals with complete dentures. The adhesives were generally well tolerated.

## Supporting information


**Figure S1.** Summary of responses to questionnaires by treatment group (modified Intent‐to‐Treat population)
**Figure S2.** Summary of responses to denture removal questionnaire (completed by participant) and questionnaire for site staff cleaning the denture by treatment group (modified Intent‐to‐Treat population)
**Table S1.** Summary of responses to product ooze questionnaire by treatment group (modified Intent‐to‐Treat population)
