# Supplementary material for: A randomised bite force study assessing two currently marketed denture adhesive products compared with no‐adhesive control
Source: Clin Exp Dent Res. 2019 May 14;5(3):276–83. doi: 10.1002/cre2.182 (PMC6585581; doi:10.1002/cre2.182)
Supplement: Supplementary file 1 — Figure S1. Summary of responses to questionnaires by treatment group (modified Intent‐to‐Treat population) Figure S2. Summary of responses to denture removal questionnaire (completed by participant) and questionnaire for site staff cleaning the denture by treatment group (modified Intent‐to‐Treat population) Table S1. Summary of responses to product ooze questionnaire by treatment group (modified Intent‐to‐Treat population) [file CRE2-5-276-s001.docx]

**SUPPLEMENTARY MATERIALS**

**SUPPLEMENTARY APPENDIX S1: Questionnaires**

*Product ooze questionnaire (completed immediately after the 0.5h bite force measurement)*

1. Did any excess denture adhesive OOZE out from under the denture after inserting in the mouth? *Yes/No.* If ‘yes’, proceed to answer Question 2.
2. If you did experience excess denture adhesive oozing out, how long after inserting your denture did this happen? *0 = immediately; 1 = less than 10 minutes; 2 = 10 to 20 minutes; 3 = 20 to 30 minutes.*

*Sensory questionnaire (completed immediately after the 1h bite force measurement, with questions pertaining to the maxillary denture only)*

1. How would you rate your OVERALL OPINION of the denture adhesive? *1 = dislike extremely; 2 = dislike moderately; 3 = dislike slightly; 4 = neither like nor dislike; 5 = like slightly; 6 = like moderately; 7 = like extremely.*
2. How much did you LIKE/DISLIKE the TASTE of the denture adhesive? *1 = dislike extremely; 2 = dislike moderately; 3 = dislike slightly; 4 = neither like nor dislike; 5 = like slightly; 6 = like moderately; 7 = like extremely.*
3. How would you describe the STRENGTH of the TASTE of the denture adhesive? *1 = no flavour at all; 2 = barely detectable; 3 = weak; 4 = moderate; 5 = strong; 6 = very strong; 7 = strongest flavour imaginable.*
4. How would you describe the TEXTURE of the dental adhesive?
   1. Smooth? *1 = not at all; 2 = slightly; 3 = moderately; 4 = very; 5 = extremely*
   2. Oily? *1 = not at all; 2 = slightly; 3 = moderately; 4 = very; 5 = extremely*
   3. Gritty? *1 = not at all; 2 = slightly; 3 = moderately; 4 = very; 5 = extremely*
   4. Soft? *1 = not at all; 2 = slightly; 3 = moderately; 4 = very; 5 = extremely*
5. How much did you feel that the adhesive was providing a CUSHIONING EFFECT on your gums? *1 = not cushioning at all; 2 = a slight amount of cushioning; 3 = a moderate amount of cushioning; 4 = a lot of cushioning; 5 = extremely cushioning.*
6. How well did you feel that the adhesive ADAPTS to the unique shape of your gums? *1 = doesn’t adapt at all; 2 = adapts slightly; 3 = adapts moderately well; 4 = adapts very well; 5 = adapts extremely well.*
7. How much did you AGREE or DISAGREE with the following statements about the adhesive?
   1. Provides a feeling of COMFORT when wearing the denture? *1 = extremely disagree; 2 = very much disagree; 3 = moderately disagree; 4 = neither agree nor disagree; 5 = moderately agree; 6 = very much agree; 7 = extremely agree.*
   2. The adhesive provides COVERAGE over my gums? *1 = extremely disagree; 2 = very much disagree; 3 = moderately disagree; 4 = neither agree nor disagree; 5 = moderately agree; 6 = very much agree; 7 = extremely agree.*

*Denture removal questionnaire (completed immediately after the 12h bite force measurement and denture removal)*

1. How EASY was it to REMOVE the denture from the mouth? *1 = not at all easy; 2 = slightly easy; 3 = moderately easy; 4 = very easy; 5 = extremely easy.*
2. How EASY was it to REMOVE ANY RESIDUAL DENTURE ADHESIVE from the mouth? *1 = not at all easy; 2 = slightly easy; 3 = moderately easy; 4 = very easy; 5 = extremely easy.*
3. How much do you AGREE or DISAGREE with the following statements about the adhesive?
   1. Provides a feeling of COMFORT when wearing the denture. *1 = extremely disagree; 2 = very much disagree; 3 = moderately disagree; 4 = neither agree or disagree; 5 = moderately agree; 6 = very much agree; 7 = extremely agree.*
   2. The adhesive provides COVERAGE over my gums. *1 = extremely disagree; 2 = very much disagree; 3 = moderately disagree; 4 = neither agree or disagree; 5 = moderately agree; 6 = very much agree; 7 = extremely agree.*
4. Please describe what you LIKED or DISLIKED about the denture adhesive [*free text answer*].
5. How EASY was it to SQUEEZE OUT the adhesive from the tube? *1 = not at all easy; 2 = slightly easy; 3 = moderately easy; 4 = very easy; 5 = extremely easy.* [Note that participants were give a tube of the study product and asked to squeeze the adhesive onto a piece of paper as per the strips that appear on the product application].

*Questionnaire for site staff cleaning the denture (completed after the 12h bite force measurement before returning the denture to the participant at the end of the treatment phase)*

1. How EASY was it to REMOVE ANY RESIDUAL DENTURE ADHESIVE from the denture? *1 = not at all easy; 2 = slightly easy; 3 = moderately easy; 4 = very easy; 5 = extremely easy.*

**Supplementary Figure S1.** Summary of responses to questionnaires by treatment

group (modified Intent-to-Treat population)

**
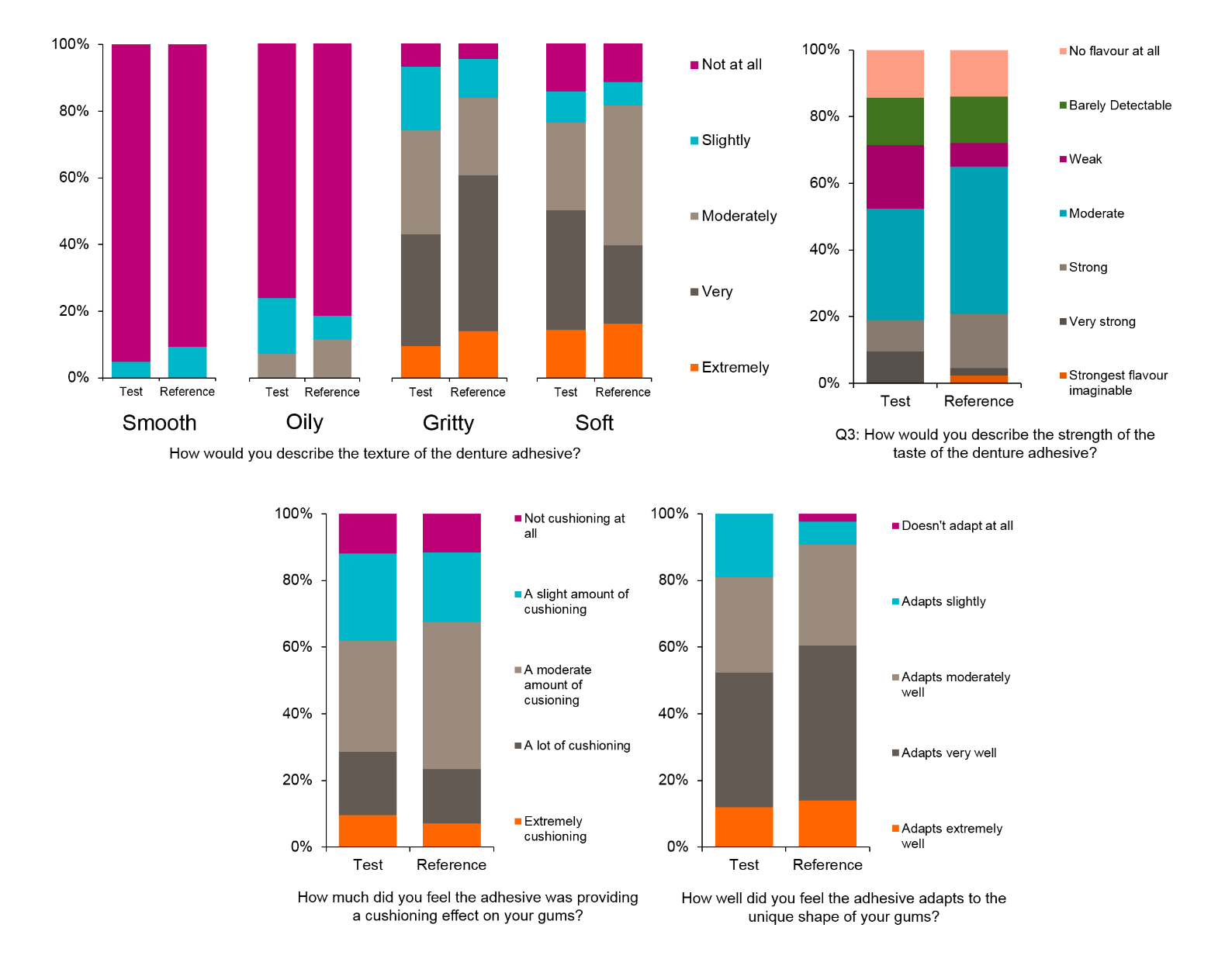
**

**Supplementary Figure S2.** Summary of responses to denture removal questionnaire (completed by participant) and questionnaire for site staff cleaning the denture by treatment group (modified Intent-to-Treat population)

**
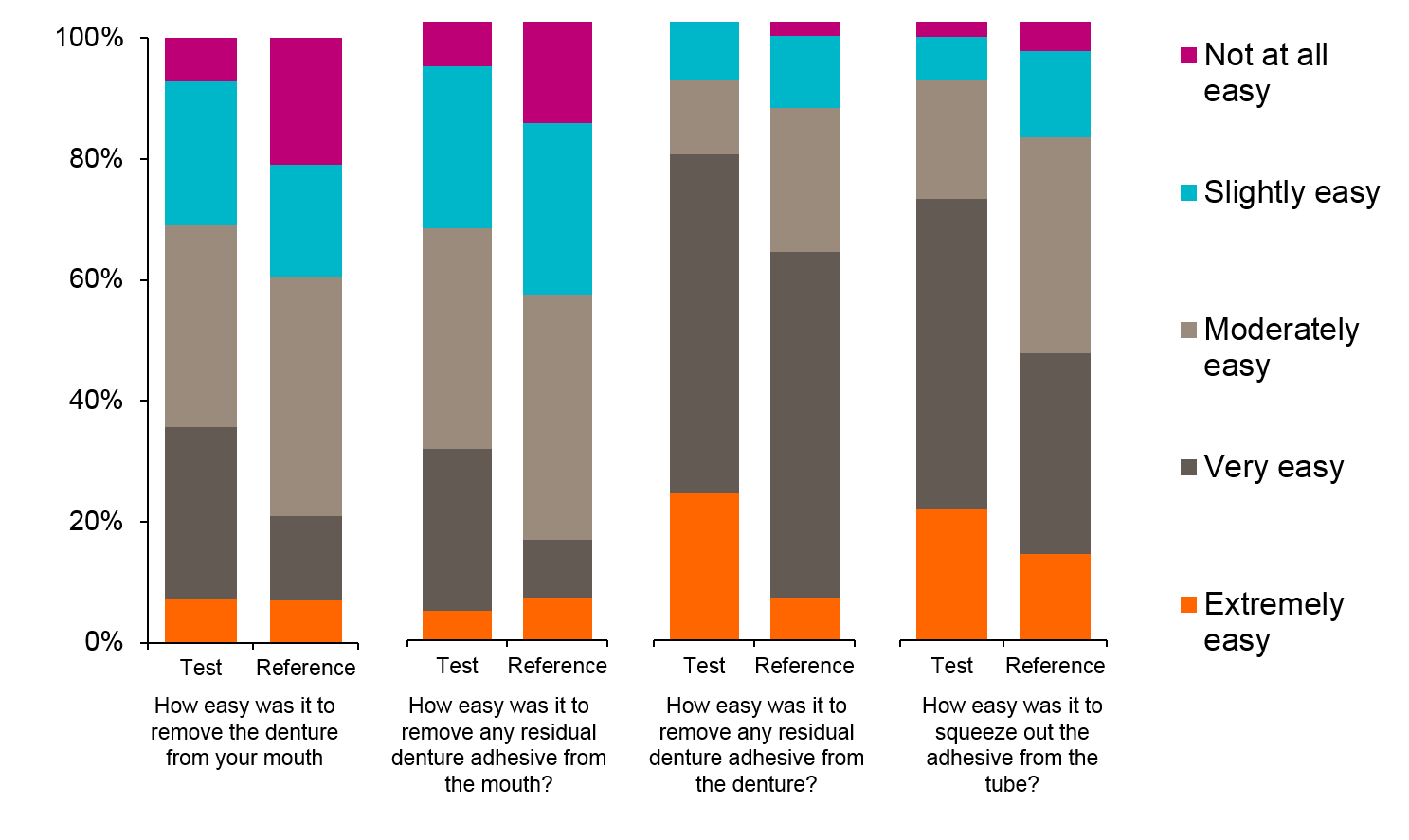
**

**Supplementary Table S1.** Summary of responses to product ooze questionnaire by treatment

group (modified Intent-to-Treat population)

|  | **Test adhesive** | **Reference adhesive** | |
| --- | --- | --- | --- |
| **Did any excess denture adhesive ooze from underneath the denture after inserting into the mouth? (n, %)** | | |  |
|  | **(n = 44)** | **(n = 44)** | |
| **Yes** | 9 (21.4) | 2 (4.7) | |
| **No** | 33 (78.6) | 41 (95.3) | |
| **Missing** | 2 | 1 | |
| **If you did experience oozing out, how long after inserting your denture did this happen? n (%)** | | |  |
|  | **(n = 9)^†^** | **(n = 2)^†^** | |
| **Immediately** | 3 (33.3) | 0 | |
| **<10 minutes** | 3 (33.3) | 2 (100) | |
| **10–20 minutes** | 3 (33.3) | 0 | |
| **20–30 minutes** | 0 | 0 | |

**^†^**n = participants who answered ‘yes’ to question 1.
